# Urogenital schistosomiasis transmission on Unguja Island, Zanzibar: characterisation of persistent hot-spots

**DOI:** 10.1186/s13071-016-1847-0

**Published:** 2016-12-16

**Authors:** Tom Pennance, Bobbie Person, Mtumweni Ali Muhsin, Alipo Naim Khamis, Juma Muhsin, Iddi Simba Khamis, Khalfan Abdallah Mohammed, Fatma Kabole, David Rollinson, Stefanie Knopp

**Affiliations:** 1 Wolfson Wellcome Biomedical Laboratories, Department of Life Sciences, Natural History Museum, Cromwell Road, London, SW7 5BD UK; 2 Department of Pathology and Pathogen Biology, Centre for Emerging, Endemic and Exotic Diseases, Royal Veterinary College, University of London, Hawkshead Lane, Hatfield, Hertfordshire AL9 7TA UK; 3 Independent Consultant, Schistosomiasis Consortium for Operational Research and Evaluation, University of Georgia, Athens, GA USA; 4 Zanzibar Neglected Tropical Diseases Programme, Ministry of Health, P.O. Box 236, Zanzibar Town, Unguja United Republic of Tanzania; 5 Swiss Tropical and Public Health Institute, Socinstrasse 57, P.O. Box, CH–4002 Basel, Switzerland; 6 University of Basel, Petersplatz 1, CH–4003 Basel, Switzerland

**Keywords:** *Bulinus globosus*, Cercariae, Control, Elimination, Hot-spot, *Schistosoma haematobium*, Snail, Safe water, Urogenital schistosomiasis, Zanzibar

## Abstract

**Background:**

Elimination of urogenital schistosomiasis transmission is a priority for the Zanzibar Ministry of Health. Preventative chemotherapy together with additional control interventions have successfully alleviated much of the disease burden. However, a persistently high *Schistosoma haematobium* prevalence is found in certain areas. Our aim was to characterise and evaluate these persistent “hot-spots” of transmission and reinfection in comparison with low-prevalence areas, to support the intervention planning for schistosomiasis elimination in Zanzibar.

**Methods:**

Prevalences of *S. haematobium* were annually determined by a single urine filtration in schoolchildren from 45 administrative areas (shehias) in Unguja in 2012, 2013 and 2014. Coverage data for biannual treatment with praziquantel were available from ministerial databases and internal surveys. Among the 45 shehias, five hot-spot (≥ 15 % prevalence) and two low-prevalence (≤ 5 %) shehias were identified and surveyed in mid-2014. Human-water contact sites (HWCSs) and the presence of *S. haematobium*-infected and uninfected *Bulinus globosus*, as well as safe water sources (SWSs) and their reliability in terms of water availability were determined and mapped.

**Results:**

We found no major difference in the treatment coverage between persistent hot-spot and low-prevalence shehias. On average, there were considerably more HWCSs containing *B. globosus* in hot-spot than in low-prevalence shehias (*n* = 8 *vs n* = 2) and also more HWCSs containing infected *B. globosus* (*n* = 2 *vs n* = 0). There was no striking difference in the average abundance of SWSs in hot-spot and low-prevalence shehias (*n* = 45 *vs n* = 38) and also no difference when considering SWSs with a constant water supply (average: 62 % *vs* 62 %). The average number of taps with a constant water supply, however, was lower in hot-spot shehias (*n* = 7 *vs n* = 14). Average distances from schools to the nearest HWCS were considerably shorter in hot-spot shehias (*n* = 229 m *vs n* = 722 m).

**Conclusion:**

The number of HWCSs, their infestation with *B. globosus* and their distance to schools seem to play a major role for a persistently high *S. haematobium* prevalence in children. In addition to treatment, increasing access to reliably working taps, targeted snail control at HWCSs near schools and enhanced behaviour change measures are needed to reduce prevalences in hot-spot areas and to finally reach elimination.

**Trial registration:**

ISRCTN48837681.

**Electronic supplementary material:**

The online version of this article (doi:10.1186/s13071-016-1847-0) contains supplementary material, which is available to authorized users.

## Background

Historically, the Zanzibar islands (Unguja and Pemba) that are part of the United Republic of Tanzania have been identified as ‘model islands’ for implementing and assessing the effectiveness of multiple infectious disease control and elimination programmes in sub-Saharan Africa. For example, due to successful control efforts over the past decades, the number of malaria cases has drastically declined [1, 2], the Tsetse fly has disappeared [3], and the transmission of lymphatic filariasis has been reduced to very low levels [4, 5]. Also urogenital schistosomiasis that imposed a considerable public health problem and formerly occurred with a very high prevalence on both islands [6–11], is now targeted for elimination [12–14].

In 2011, the Zanzibar Elimination of Schistosomiasis Transmission (ZEST) alliance was formed to work towards the elimination of urogenital schistosomiasis [12]. Moreover, in 2012, the World Health Organization (WHO) set the goal to interrupt transmission of schistosomiasis in selected countries of the African region by 2025 [15], and Zanzibar has been mentioned as a place where concerted efforts for elimination have begun [16]. Since 2012, the Zanzibar Ministry of Health has been carrying out biannual preventive chemotherapy (PC) assisted by the Schistosomiasis Control Initiative (SCI) and WHO. To address research questions related to the elimination of urogenital schistosomiasis on Zanzibar, an operational research project supported by the Schistosomiasis Consortium for Operational Research and Evaluation (SCORE) has been implemented in selected communities on Unguja and Pemba islands since November 2011 [12, 13]. The cluster randomized trial has three study arms to assess the impact of (i) biannual PC, (ii) biannual PC plus snail control, and (iii) biannual PC plus behaviour change interventions, respectively, on *Schistosoma haematobium* prevalences and infection intensities [13].

The baseline parasitological survey conducted within the SCORE operational research trial revealed an overall *S. haematobium* prevalence of 7 % and 4 %, in the school-aged and adult communities in Zanzibar, respectively [14]. Mean infection intensities at baseline were 5 eggs and 0.7 eggs in 10 ml urine, respectively. Despite these very low overall endemicity levels, some communities with a considerably higher prevalence (up to 32 %) were identified at baseline [14]. In 2014, after all interventions had been implemented for two years, some of these communities had maintained a persistently high or even increasing prevalence, signifying high levels of ongoing transmission and reinfection, and thus referred to here as “persistent hot-spot” areas.

Persistent hot-spots following control strategies for schistosomiasis have been identified not only on Zanzibar [6, 10, 17, 18] but also in other countries such as Morocco [19], Kenya [20–22] and China [23]. Multiple factors that can significantly increase the risk of infection with schistosomes might be important drivers for the existence and persistence of schistosomiasis transmission hot-spot areas and their resilience to PC and other intervention measures such as snail control and health education. These risk factors include living in close proximity to a freshwater body containing intermediate host snail species [17, 21, 24], frequent and intense contact of humans with natural freshwater [17, 25], lack of drinking water sources and latrines in schools [25] and the construction of agricultural water schemes [26]. Also “super-spreaders”, potentially untreated or treated and then re-infected people that harbour heavy infections, might contribute to the perpetuation or resurgence of transmission [27, 28].

To target control interventions adequately, to reduce the prevalence and infection intensity in all areas and to finally reach elimination of urogenital schistosomiasis transmission across Zanzibar, key factors contributing to the persistence of hot-spots need to be identified. In the study presented here we aimed (i) to identify persisting *S. haematobium* transmission hot-spots and compare them with low-prevalence areas according to prevalence data obtained in the annual parasitological surveys of the SCORE project carried out in 2012, 2013 and 2014 (ii) to locate, map and count human-water contact sites (HWCSs) and safe water sources (SWSs) in selected persistent hot-spot and low-prevalence areas, (iii) to collect intermediate host snails (*Bulinus globosus*) from surveyed HWCSs, to ‘shed’ them (i.e. induce cercarial emergence) under laboratory conditions and to determine the prevalence of *S. haematobium* infections in snails at each HWCS, and (iv) to compare features of persistent hot-spot and low-prevalence areas to better target future interventions.

## Methods

### Study area and population

Unguja island, where this work was conducted, is divided into six districts that are further divided into 210 smaller administrative areas [29], referred to as shehias. A community leader, the sheha, locally governs each shehia. On Unguja, the average area of a shehia is 9.5 km^2^. A shehia can contain several villages, which can vary in population size and household number.

Transmission of urogenital schistosomiasis on Unguja is geographically restricted by the presence of the intermediate host snail species *B. globosus*, which is only found in the northern, western and central parts of the island but not in the South [30, 31]. Our study hence focussed on shehias located in areas, where *B. globosus* is endemic [32].

The study was conducted in June and July 2014, coinciding with the tail end of the long rainy season (Masika rains), which usually lasts from March till June. Hence, during the study period, heavy rain was rare and day temperatures were moderate at 23–28 °C.

### Selection criteria for hot-spot and low-prevalence shehias

Among the 45 shehias included in the SCORE project on Unguja [13], all shehias with a *S. haematobium* infection prevalence of ≥ 15 % in 9–12 year-old schoolchildren in at least one of the three cross-sectional parasitological surveys conducted in primary schools of the shehias in 2012, 2013 and 2014, respectively, were considered as persistent hot-spots. Shehias with a prevalence of ≤ 5 % in schoolchildren in all three parasitological surveys were considered as low-prevalence shehias. For operational reasons, a rigorous mapping exercise and snail survey was only feasible in a limited number of areas. Hence, shehias were further selected based on their attribution to intervention arms in the SCORE study and based on their location and geography.

### Collection and examination of urine samples within the SCORE study

The *S. haematobium* prevalence in children aged 9–12 years is monitored annually in the 45 study schools on Unguja as part of the SCORE project [13]. Prior to the beginning of this study, parasitological surveys had been conducted in 2012, 2013 and 2014. Details of the survey procedure, selection and randomization of children and examination of urine samples for *S. haematobium* infection in the laboratory are described in detail in the published study protocol [13].

### Assessment of praziquantel treatment and coverage

In an effort to eliminate urogenital schistosomiasis in Zanzibar, the whole eligible population on Unguja and Pemba, with the exception of children below the age of three years, pregnant women and severely sick persons, is treated biannually with praziquantel since April 2012 [33]. Hence, before the implementation of this study in June and July 2014, four community-wide treatment (CWT) rounds had been conducted by the Zanzibar Ministry of Health in April and November 2012 and in June and November 2013. In November 2013, children attending primary schools were supposed to receive praziquantel in their school and not in their community.

Coverage for every CWT round was reported by the Zanzibar Ministry of Health, which collected records from community drug distributors (CDDs). Coverage for the school-based treatment (SBT) round was reported by the Ministry of Health in line with records provided by the schoolteachers and its own staff. Moreover, a post-treatment survey was conducted within the SCORE study for the CWT and SBT rounds that were conducted in November 2013 [33].

### Identification and characterization of HWCSs

Several types of HWCSs occur in Unguja: ponds (defined as a small standing body of water), rivers (defined as a water body with a current that leads to a lake or the sea), streams (defined as body of water with a current that eventually joins to a river) and rice paddies (defined as flooded piece of land used for cultivating semiaquatic rice). All of these HWCSs can potentially act as suitable environments for *B. globosus* [34]. Human-water contact sites were defined as points where people can gain access to open freshwater bodies. The sites were located with the help of the sheha, assistant sheha, or any village member familiar with the geography of the shehia and mapped with a handheld Garmin GPSMAP 62sc device (Garmin, Kansas City, USA). Each HWCS was investigated for the presence of intermediate host snails as described below. Moreover, physical and chemical water characteristics (temperature, pH, conductivity and total dissolved solids) of the HWCSs and ecological characteristics such as substrates and vegetation were assessed and recorded as described elsewhere in more detail [13]. Additionally and as part of the HWCS characterisation, human activities that bear a risk for *S. haematobium* infection and transmission (e.g. swimming and washing clothes) were determined by observation of evidence and recorded.

### Collection of intermediate host snails

At each identified HWCS, a snail survey was conducted aiming to identify all snail species present and to specifically collect *Bulinus* snails. At each identified site, two collectors searched for snails of all species for 15 min and over no more than a 15 m^2^ area. Borders of the water body and vegetation, where snails were most likely to be found, were more intensively searched than other areas. In rivers and streams snail collectors moved up- and downstream from the access point whenever possible to target slow flowing regions that provide a more suitable environment for snails [35]. Snails were primarily searched for by hand, but a snail scoop was also used to reach under deeper vegetation or sites with restricted access. All snails were identified to at least the genus level, but only *B. globosus* and *B. forskalii* were removed from HWCSs, placed in screw top plastic containers with freshwater from the site and taken to the laboratory for cercariae shedding. The snail species and additional information about the habitat, such as vegetation and foliage at the site, was recorded. Snail collections were only conducted on clear days, as rain before collection times could affect the positioning of snails in the water and alter the ‘catch’ size.

### Cercariae shedding and collection

All collected *Bulinus* snails were examined for cercariae shedding in the laboratory of the Zanzibar Neglected Tropical Diseases Programme in Zanzibar Town. Snails were examined following methods described by Allan et al. [35]. Cercariae of *S. haematobium* were identified by an experienced microscopist and transferred onto a Whatman FTA card (Whatman, Part of GE Healthcare, Florham Park, USA) for long term deoxyribonucleic acid (DNA) storage using a micropipette (20 μl Gilson PIPETMAN Classic, Gilson Inc, Wisconsin, USA) set to 3.5 μl. The FTA card was labelled with collection details and stored at the Schistosomiasis Collection at the Natural History Museum (SCAN) in London, UK [36]. After shedding, all infected snails were placed in 70 % ethanol in collection jars for future molecular examination at the Natural History Museum (NHM), London.

### Identification and characterization of SWSs

Public taps, wells and water pumps were considered as SWSs. In each shehia, SWSs were located with the help of the sheha, assistant sheha, or any village member familiar with the shehia and mapped with a handheld Garmin GPSMAP 62sc device (Garmin, Kansas City, USA). The availability of clean water was assessed by turning the tap, pumping the pump or checking for groundwater in wells and recorded. If water was not present when the SWS was visited, an inhabitant of a nearby house was questioned whether water was ever available from that point, when it was usually available and whether it was clean. Additional information on water availability was obtained by asking shehia inhabitants about the seasonality and day-to-day readiness of the water from each source.

### Distances

Digital maps of the United Republic of Tanzania (including Zanzibar) were obtained from the International Livestock Research Institute (www.ilri.org) and shape files for Zanzibar were provided by the Health Management Information System of the Revolutionary Government of Zanzibar (www.hmis.zanhealth.go.tz). ArcGIS (version 10.2.2) (Esri, California, USA) was used to create maps displaying locations of villages, schools, HWCSs and SWSs in each investigated shehia. The World Geodic System 1984 (version WGS 84 revised in 2004) was used for mapping all coordinates. Coordinates were collected in degrees, minutes and seconds (DMS) and subsequently converted into decimal degrees (DD). The ‘measure’ tool on ArcGIS was used to determine distances between primary schools and the closest HWCSs or SWSs, respectively.

### Data management and analysis

Field data were recorded on paper collection forms and subsequently entered into a Microsoft Excel 2013 (Version 14.0.0.0) database. All data were analysed using STATA/MP 14.1 (StataCorp, College Station, USA).

The *S. haematobium* prevalence was calculated from data collected during the parasitological surveys in 2012, 2013 and 2014. Treatment coverage was defined as the proportion of individuals among the total population that had received praziquantel tablets. With regard to the data obtained from the Ministry of Health, coverage was calculated as the percentage of people who had received tablets among the total number of people as recorded by the CDDs or teachers. In the post-treatment survey conducted in the SCORE schools and communities in early 2014, we calculated coverage as the proportion of pupils and adults, respectively, who received praziquantel among those who were interviewed and included into our analysis [33]. SWSs were classified as either ‘always’ available if water could be collected from the SWS throughout the year and at all times of the day, or ‘not always’ available if water could only be collected during certain seasons or times of the day. Univariable and multivariable regression analyses were used to investigate potential associations, expressed in odds ratios (ORs), between *B. globosus* presence or infected *B. globosus* presence as outcomes and water chemistry, ecological characteristics, water body types, presence of other snail species and behavioural activities as explanatory variables. Univariable regression was also used to explore an association between SWS type and water availability. Explanatory variables for the multivariable regression were all variables that were present in at least 5 % of observations. Multivariable regression models were conducted for: (i) *B. globosus* presence and water chemistry; (ii) *B. globosus* presence and ecological characteristics; (iii) *B. globosus* presence and water body type; (iv) *B. globosus* presence and presence of other snails species; (v) *B. globosus* presence and human behavioural activities observed at HWCSs; (vi) infected *B. globosus* presence and water chemistry; and (vii) infected *B. globosus* presence and human behavioural activities observed at HWCSs. Multivariable regression models allowed for clustering and removed non-predicting covariates up to a significance level of 0.2 in a stepwise procedure.

## Results

### Selected persistent hot-spot and low-prevalence shehias

**Fig. 1:**
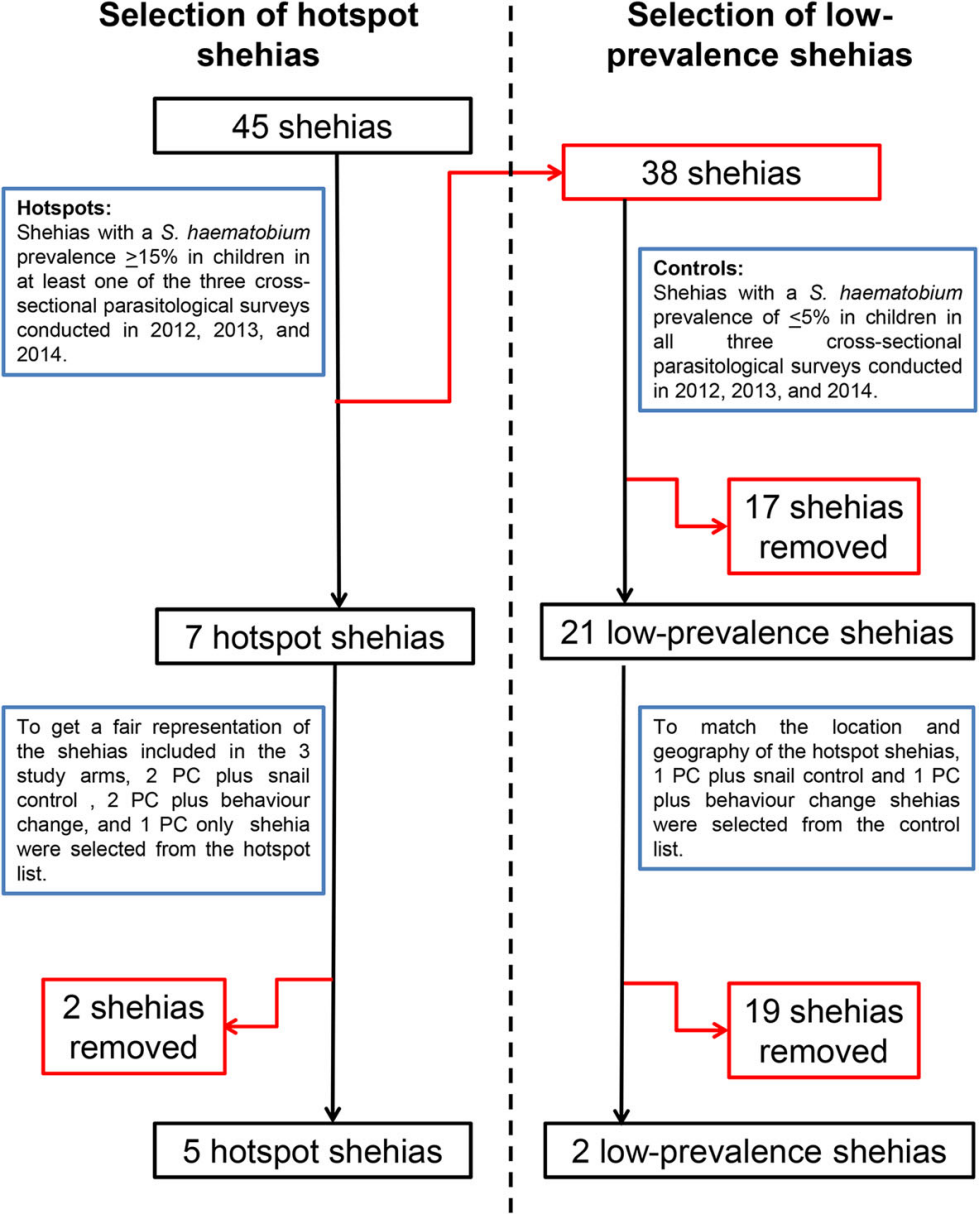
Flowchart showing the inclusion procedure for persistent hot-spot and low-prevalence shehias in Unguja

**Fig. 2:**
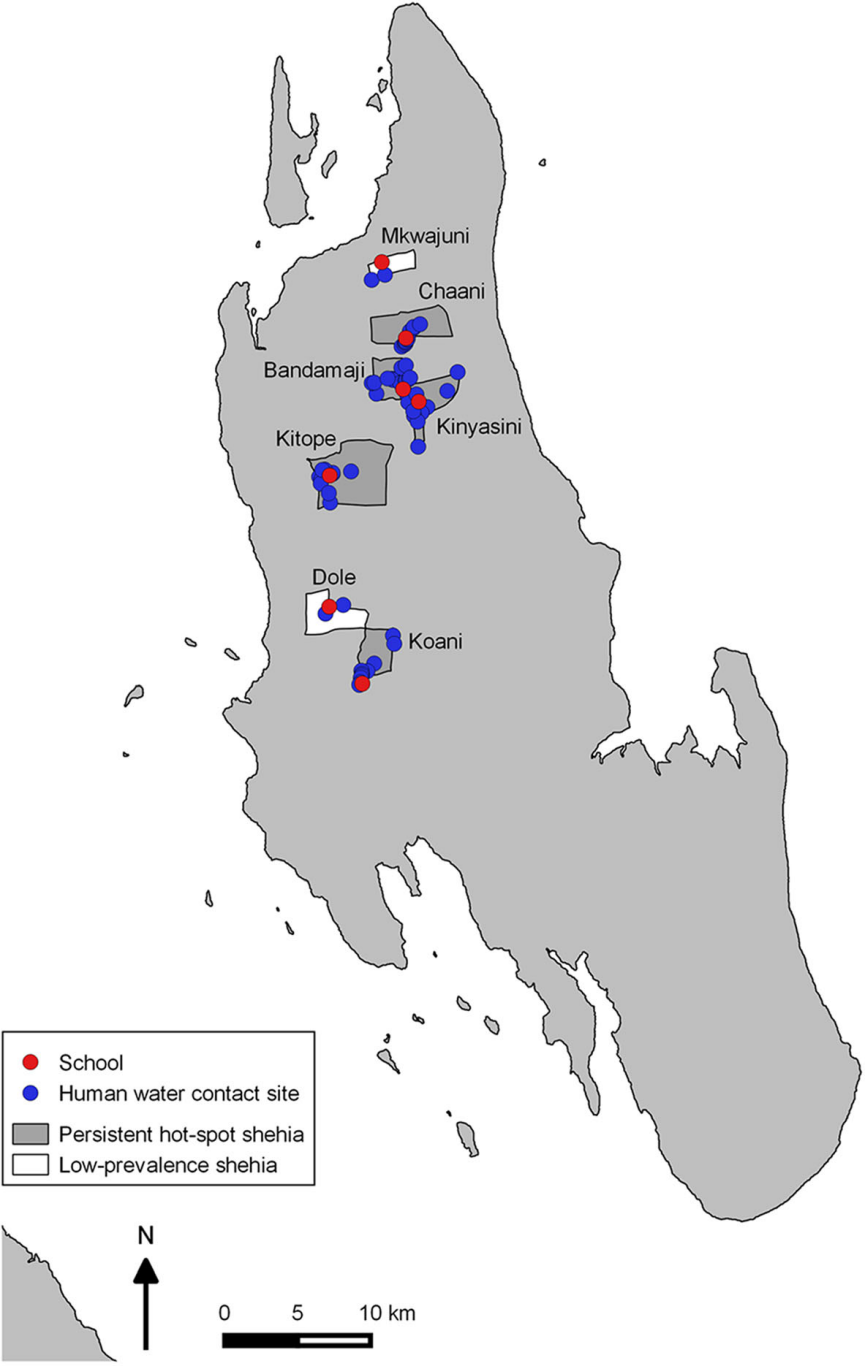
Map of Unguja Island, Zanzibar, showing the location of selected persistent hot-spot and low-prevalence shehias

As shown in Fig. 1, among the 45 shehias that are part of the SCORE project in Unguja, seven shehias fit the criteria for persistent hot-spots and 21 shehias fit the criteria for low-prevalence shehias. For operational feasibility, a total of five persistent hot-spot shehias and two low-prevalence shehias were included in the study. The following five persistent hot-spot shehias were selected: Bandamaji, Chaani, Kinyasini, Kitope and Koani. The following two shehias were selected as low-prevalence shehias: Dole and Mkwajuni. The location of each of the surveyed shehias in Unguja is shown in Fig. 2, along with point locations of schools and HWCSs.

### Prevalence of *S. haematobium* in persistent hot-spot and low-prevalence shehias

Table 1 shows the prevalence of *S. haematobium* in children visiting the primary schools located in any of the seven study shehias, stratified by survey year. The average *S. haematobium* prevalence across all three survey years in 9–12 years old schoolchildren was 20.0 % (95 % CI: 18.1–22.0) in the five persistent hot-spot shehias and 0.78 % (95 % CI: 0.0–1.5) in the two low-prevalence shehias. Schoolchildren in Koani shehia had the highest average prevalence of 26.4 % considering all surveys conducted in schools between 2012 and 2014 and also had the highest single-year prevalence of 37.6 % in 2013.

**Table 1:** Prevalence of *S. haematobium* in schoolchildren in persistent hot-spot and low-prevalence shehias in Unguja

	Population size (2014)^a^	2012 (1st year students)		2012 (9–12 year-old students)		2013 (9–12 year-old students)		2014 (9–12 year-old students)	
		*n*/*N* ^b^	%^c^	*n*/*N*	%	*n*/*N*	%	*n*/*N*	%
Persistent hot-spot shehias									
Bandamaji	1273	5/44	11.4	16/94	17.0	24/89	27.0	32/117	27.4
Kinyasni	3302	24/100	24.0	21/105	20.0	20/108	18.5	37/112	33.0
Chaani	3666	8/77	10.4	6/131	4.6	35/113	31.0	12/119	10.1
Koani	3083	15/102	14.7	15/94	16.0	41/109	37.6	27/111	24.3
Kitope	2526	24/96	25.0	12/89	13.5	9/111	8.1	16/112	14.3
Low-prevalence shehias									
Dole	3015	0/36	0.0	0/49	0.0	1/58	1.7	0/62	0.0
Mkwajuni	4792	1/97	1.0	0/118	0.0	2/115	1.7	1/111	0.9

^a^Shehia population sizes were estimated by multiplying the population size recorded in the 2012 Population and Housing census [29] by the annual growth rate (2.8 %)

^b^ *N* = number of children sampled; *n* = number of children infected

^c^% = percentage of children infected with *S. haematobium*

### Treatment coverage in persistent hot-spot and low-prevalence shehias

As indicated in Table 2, the coverage achieved in the four CWT rounds and the SBT round varied considerably per round and per shehia. While the coverage from rounds 1 and 2 conducted in 2012 was reported to be ≥ 75 % in all persistent hot-spot shehias except Bandamaji, a ≥ 75 % coverage in round 3 was only reported for Koani and in round 4 for Koani and Kinyasini. In the low-prevalence shehias, a ≥ 75 % coverage was reported for both Dole and Mkwajuni in rounds 1 and 2 and for Mkwajuni in round 4.

**Table 2:** Treatment coverage in persistent hot-spot and low-prevalence shehias in Unguja

	CWT 1 in April 2012	CWT 2 in November 2012	CWT 3 in June 2013	CWT 4 in November 2013	SBT in November 2013	Post-CWT 4	Post-SBT
	% received tablets	% received tablets	% received tablets	% received tablets	% received tablets	% received tablets	% received tablets
Persistent hot-spot shehias							
Bandamaji	73.2	79.8	56.5	57.1	68.8	76.5	88.6
Kinyasini	88.0	81.1	51.0	75.1	31.9	72.0	89.2
Chaani	89.0	86.0	44.0	70.3	93.0	82.6	94.7
Koani	78.3	93.1	78.3	77.0	77.8	72.0	86.7
Kitope	94.5	92.5	72.7	70.5	52.6	46.0	84.2
Overall coverage	87.8	86.7	58.2	71.7	65.6	69.6	88.8
Low-prevalence shehias							
Dole	90.3	87.3	74.9	65.3	Not listed	69.6	94.2
Mkwajuni	94.0	78.9	58.9	79.1	Not listed	53.1	0.0
Overall coverage	92.7	81.6	67.2	73.0		61.1	34.4

*Abbreviations*: *CWT* community-wide treatment, *SBT* school-based treatment

A SBT coverage was only reported by the Ministry of Health for the five persistent hot-spot shehias, but not for the two low-prevalence shehias. The school-based reported coverage was ≥ 75 % in Chaani and Koani schools.

The SCORE coverage survey conducted for the CWT round 4 indicated an observed coverage of ≥ 75 % only in the persistent hot-spot shehias Bandamaji and Chaani. Moreover, a coverage of ≥ 75 % was observed in all schools in the five persistent hot-spot shehias and in the school in the low-prevalence shehia in Dole. Surveyed children in the school in the low-prevalence shehia Mkwajuni had not received treatment.

### Human-water contact sites in persistent hot-spot and low-prevalence shehias

**Fig. 3:**
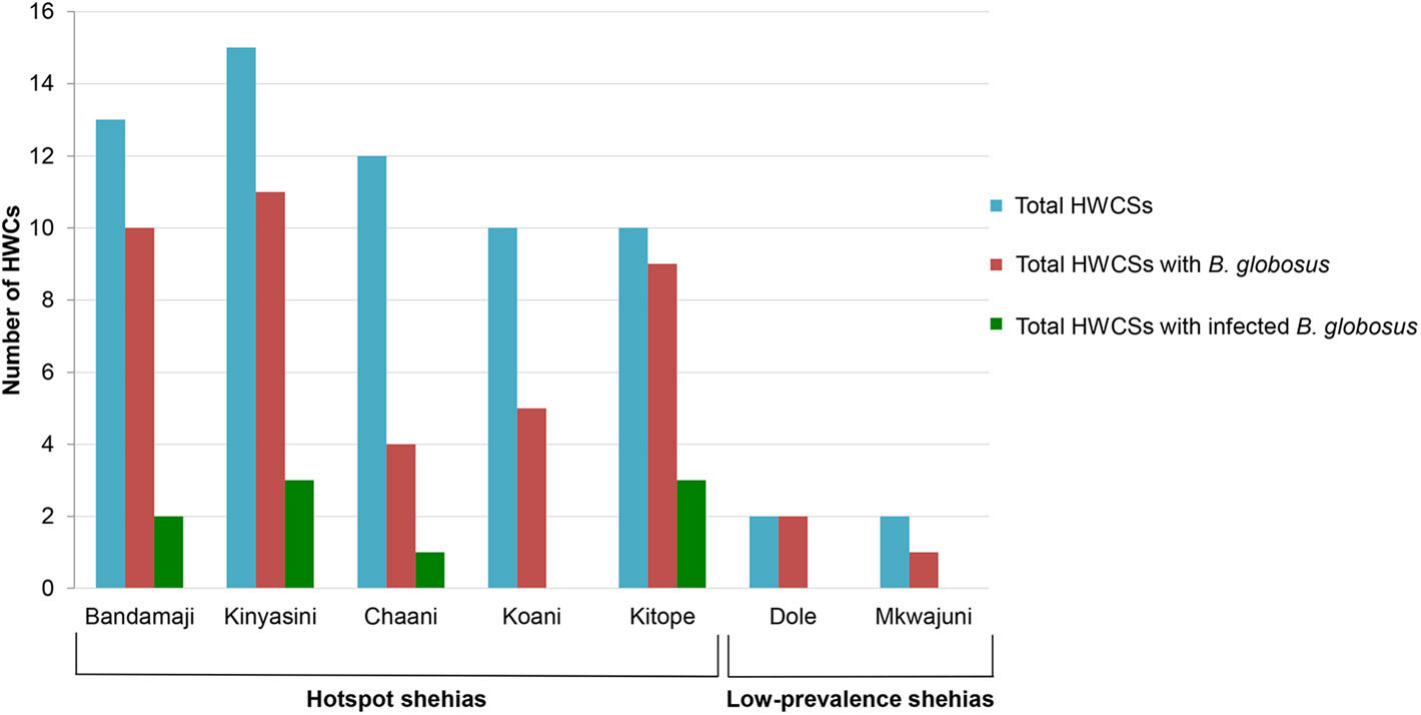
Number of human-water contact sites in persistent hot-spot and low-prevalence shehias in Unguja

A total of 66 HWCS were surveyed across the five persistent hot-spot shehias and two low-prevalence shehias in Unguja. Among those, 56 were permanent and 10 were seasonal HWCS. As shown in Fig. 3, the number of HWCSs ranged from 10 to 15 in persistent hot-spot shehias, while only two HWCSs were located in each of the two low-prevalence shehias. Among the persistent hot-spot shehias, Kinyasini had the highest number of HWCSs (*n* = 15), while Koani and Kitope had the lowest number (*n* = 10). Ponds were the most common freshwater bodies and every shehia, with the exception of Chaani, had at least one HWCS at a pond. Rivers contained a lot of HWCSs in Kinyasini (*n* = 8) and Bandamaji (*n* = 3). Smaller streams included the majority of HWCSs in Chaani (*n* = 10) and Kitope (*n* = 7), but HWCSs at streams were also present in lower numbers in Kinyasini (*n* = 2) and Koani (*n* = 1). Rice paddies were only present in Koani (*n* = 1) and Chaani (*n* = 2). The four HWCSs in the low-prevalence shehias were one pond and one small stream in Mkwajuni and one pond and one rice paddy in Dole.

Water chemistry was recorded in 61 among the 66 HWCSs surveyed. The water chemistry device failed recording at two HWCSs at ponds in Bandamaji and at three HWCSs at ponds in Koani. At the 61 HWCSs surveyed, the average temperature was 26.7 °C (range: 24.1–35.8 °C), pH was 9.6 (range: 6.8–10.5), conductivity was 482.1 μS (range: 74.0–771.0 μS) and total dissolved solids was 250.8 ppm (range: 37.0–610.0 ppm). Salinity remained zero at all sites.

### Intermediate host snail collections and cercariae shedding

**Fig. 4:**
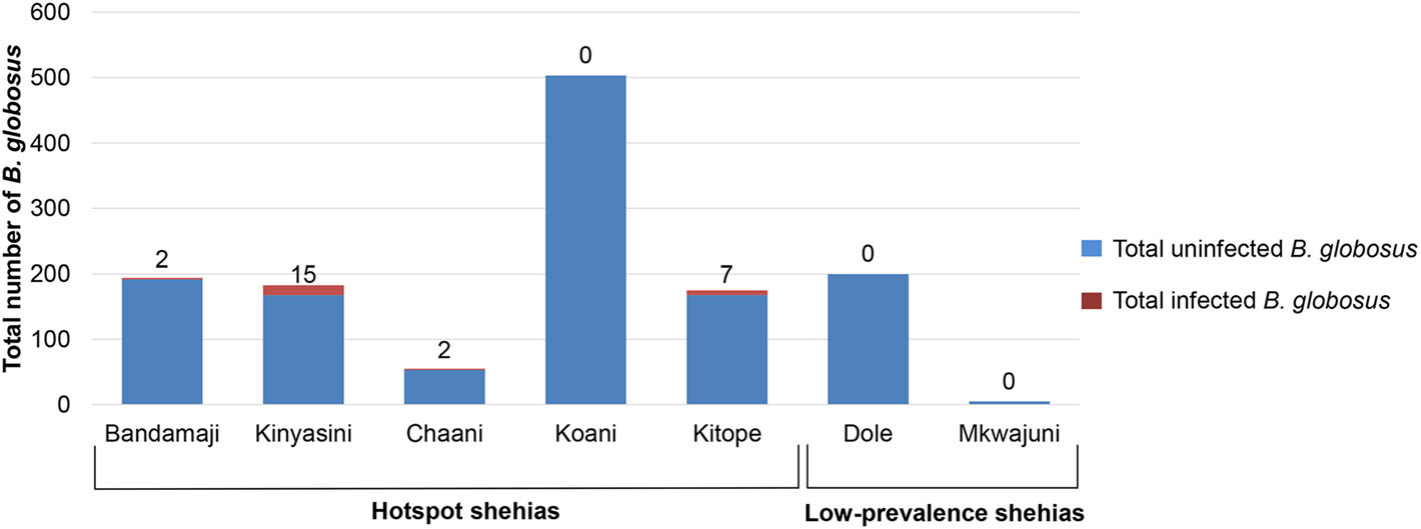
Number of *B. globosus* and *B. globosus* shedding *S. haematobium* cercariae per shehia in Unguja

As shown in Fig. 3, *B. globosus* were found in at least half of all investigated HWCSs in all surveyed shehias, with the exception of Chaani, where the intermediate host snails were found in a third of all HWCSs. In total, 1111 *B. globosus* were collected from 39 HWCSs in persistent hot-spot shehias. Among the 1111 *B. globosus*, 26 (2.3 %) were found to have a patent *S. haematobium* infection. As shown in Fig. 4, the highest number of infected snails per shehia was found in Kinyasini, where 15 (8.2 %) of *B. globosus* were infected. Among the four HWCSs in low-prevalence shehias, three contained *B. globosus*. However, none of the 205 *B. globosus* collected in the low-prevalence shehias shed cercariae. Univariable regression indicated no association between the presence of infected snails and the total number of *B. globosus* collected at each site (OR = 1.0, 95 % CI: 1.0–1.0).

*Bulinus forskalii* was collected from a total of seven HWCSs in the persistent hot-spot shehias Bandamaji, Chaani, Kinyasini and Kitope, and from two HWCSs in the low-prevalence shehia Mkwajuni. No *B. forskalii* was found to be infected with *S. haematobium*. Two other groups of snails were also common and visually identified to the genus level: *Pila* sp. was found in 66.6 % and *Cleopatra* sp. was found in 65.2 % of the surveyed HWCSs.

### Factors influencing the presence of *B. globosus* and infected *B. globosus*

Results of the univariable and multivariable regression models are provided in Additional file 1: Tables S1 and S2. Multivariable regression analyses showed that none of the investigated water chemistry or water body type characteristics were associated with the presence of *B. globosus*. With regard to ecological characteristics, only the presence of grass at HWCSs was significantly associated with the presence of *B. globosus* (OR = 4.2, 95 % CI: 1.2–14.6). The model for presence of other snail species revealed that the presence of *Pila* sp. was significantly associated with the presence of *B. globosus* (OR = 25.4, 95 % CI: 6.0–107.8).

The multivariable model including behavioural activities observed at HWCSs revealed an association of *B. globosus* presence with fishing (OR = 49.4, 95 % CI: 5.3–463.7), bathing (OR = 0.1, 95 % CI: 0.0–0.6) and swimming/playing (OR = 0.0, 95 % CI: 0.0–0.1).

The presence of *S. haematobium* infected *B. globosus* was not significantly related to any water chemistry, ecological characteristics, water body types or presence of other snail species. However, a significant association between the presence of infected *B. globosus* and the following behavioural activities was determined in a multivariable model including behavioural parameters: washing dishes (OR = 98.9, 95 % CI: 5.2–1881.4), fishing (OR = 58.3, 95 % CI: 1.3–2616.6), water collection for drinking and cooking (OR = 0.0, 95 % CI: 0.0–0.2), and clothes washing (OR = 0.2, 95 % CI: 0.0–0.9).

### Characteristics of safe water sources

Table 3 indicates that in persistent hot-spot shehias, the total number of public SWSs per shehia ranged from 16 to 61 and included wells, pumps and taps. Taps were the most common SWSs in Kitope (75.9 %) and Chaani (63.9 %). Wells were more abundant in Bandamaji (93.8 %), Koani (80.7 %) and Kinyasini (80.6 %). The number of pumps was generally low. Only in Chaani, pumps made up for 13.1 % among other SWS. Noteworthy, 90.3 %, 84.2 %, 56.3 %, 54.1 % and 20.7 % of the SWSs in Kinyasini, Koani, Bandamaji, Chaani and Kitope, respectively, had water constantly available.

**Table 3:** Abundance of public safe water sources supplying persistent hot-spot and low-prevalence shehias in Unguja

	Wells		Taps		Pumps		Other SWSs^c^		Total SWSs	
	*n*/*N* ^a^	%^b^	*n*/*N*	%	*n*/*N*	%	*n*/*N*	%	*n*/*N*	%
Persistent hot-spot shehias										
Bandamaji	8/15	53.3	0/0	–	1/1	100	0/0	–	9/16	56.3
Kinyasini	22/25	88.0	3/3	100	3/3	100	0/0	–	28/31	90.3
Chaani	3/14	21.4	23/39	59.0	7/8	87.5	0/0	–	33/61	54.1
Koani	40/46	87.0	7/10	70.0	1/1	100	0/0	–	48/57	84.2
Kitope	10/13	76.9	2/44	4.6	0/1	0.00	0/0	–	14/58	24.1
Low-prevalence shehias										
Dole	6/12	50.0	10/39	25.6	1/1	100	1/2	50.0	18/54	33.3
Mkwajuni	2/2	100.0	18/20	90.0	0/0	–	0/0	–	20/22	90.9

*Abbreviations*: *SWS* safe water source

^a^ *N* = total number of public safe water sources; *n* = number of SWSs that constantly provided water

^b^% = percentage of SWSs that constantly provided water

^c^Other SWSs included a hose and a leaking electric ground water pump in Dole

In low-prevalence shehias, the total number of public SWSs per shehia ranged from 22 to 54 and included wells, pumps and taps and in Dole also a hose and a leaking electric groundwater pump. Taps were the most common SWS in both Mkwajuni (90.9 %) and Dole (72.2 %). In the low-prevalence shehias, water was constantly available in 90.9 % of the SWSs in Mkwajuni and in 33.3 % of the SWSs in Dole.

The mean number of SWSs was 44.6 (95 % CI: 27.0–62.2) and 38.0 (95 % CI: 6.6–69.4) in persistent hot-spot and low-prevalence shehias, respectively. Water was constantly available in 61.6 % (95 % CI: 38.4–84.8) of the SWSs in persistent hot-spot shehias and in 62.0 % (95 % CI: 5.2–118.8) of the SWSs in low-prevalence shehias.

Univariable regression showed that taps had significantly lower odds of water being constantly available in persistent hot-spot shehias (OR = 0.2, 95 % CI: 0.1–0.4), but not in low-prevalence shehias (OR = 0.7, 95 % CI: 0.2–2.2). No other SWS type was significantly related to water availability.

### Distances from schools to human-water contact sites and safe water sources

Table 4 indicates the direct (i.e. straight line) distances from the primary school in each shehia to the nearest HWCS or SWS. In persistent hot-spot shehias, the average distance from primary schools to the nearest HWCS was 229 m (95 % CI: 58–400) and to the nearest HWCS containing *B. globosus* was 245 m (95 % CI: 90–400). In low-prevalence shehias, the average distance to both the nearest HWCS and the nearest HWCS with *B. globosus* was 722 m (95 % CI: -1506–2950).

**Table 4:** Distances (in metres) from schools to human-water contact sites and safe water sources

Primary school	HWCS	HWCS with *B. globosus*	HWCS with infected *B. globosus*	SWS	SWS always available
Persistent hot-spot shehias					
Bandamaji	460	460	620	95	95
Kinyasini	169	169	568	143	143
Chaani Masingini	134	148	148	54	54
Koani	152	218	–	376	376
Kitope	229	229	531	44	246
Low-prevalence shehias					
Dole	546	546	–	60	212
Mkwajuni	897	897	–	207	207

*Abbreviations*: *HWCS* nearest human-water contact site at freshwater bodies, *SWS* nearest safe water source

The average distance from primary schools to the nearest SWS was 142 m (95 % CI: -28–312) in persistent hot-spot shehias and 134 m (95 % CI: -799–1067) in low-prevalence shehias. The direct way to the nearest SWS with constant water availability was 183 m (95 % CI: 22–344) in persistent hot-spot shehias and 210 m (95 % CI: 179–241) in low-prevalence shehias.

In all shehias but Koani, the distance from the primary school to the nearest SWS and SWS with constant water flow was shorter than the distance to the nearest HWCS. In Koani, the distance from Mwera primary school to the nearest HWCS was 152 m, while the distance to the nearest SWS was 376 m.

## Discussion

Urogenital schistosomiasis transmission hot-spot areas in Zanzibar have remained resilient to PC and additional control interventions for multiple years. We aimed to better characterise persistent hot-spot shehias in Unguja to inform and improve intervention planning for schistosomiasis elimination in Zanzibar.

No major difference in the treatment coverage was found between persistent hot-spot and low-prevalence shehias. The SCORE coverage survey revealed that while the observed coverage in all targeted schools in 2013 was above the 75 % mark, the overall observed coverage for CWT in persistent hot-spot and low-prevalence shehias was 70 % and 61 %, respectively. Hence, a substantial part of the population remained untreated and potentially infected individuals might have contributed to the perpetuation of transmission in areas where intermediate host snails were present. Recent modelling work has shown that interruption of schistosomiasis transmission in moderate intensity settings is possible, if at least 75 % of school-aged children are treated annually with praziquantel and if a moderate treatment coverage in adults is reached [37]. However, heterogeneity in terms of water contact type and the sort of aquatic habitat near each village had not been considered and would require individual based stochastic models, which incorporate spatial transmission [37, 38].

Indeed, we identified considerably more HWCSs containing *B. globosus* (average: *n* = 8 *vs n* = 2) and *B. globosus* infected with *S. haematobium* (average: *n* = 2 *vs n* = 0) in persistent hot-spot than in low-prevalence shehias. *Bulinus globosus* infected with *S. haematobium* were found exclusively at HWCSs located in hot-spot but not in low-prevalence shehias. The proportion of snails with a patent infection (2 %) found in the persistent hot-spot shehias in our study, is in line with the proportion of snails collected with patent infections reported from other studies conducted in Zanzibar and elsewhere in sub-Saharan Africa [35, 39–41]. While these infection levels seem rather low considering the *S. haematobium* prevalence in children in the persistent hot-spot shehias, previous work conducted in Zanzibar has shown that the cercariae shedding method misses many prepatent infections [35]. If more advanced, molecular techniques are used for screening snails instead, it is likely to detect a considerably higher number of infected snails [35, 40, 42–44]. Rapid detection of schistosome cercariae ribosomal DNA in environmental samples using new methods could also aid in uncovering transmission sites that may have previously been missed following classic snail ‘shedding’ methods [45].

Our study also revealed that the distance to HWCSs containing intermediate host snails was shorter from schools with high *S. haematobium* prevalence than from the low-prevalence schools where the *S. haematobium* prevalence was < 5 %. Similarly, another study from Zanzibar had shown that the highest *S. haematobium* prevalence was found in village hamlets that were located in close proximity to HWCSs containing *B. globosus* and infected *B. globosus* [17]. Also in Mali, the vicinity of intermediate host snail breeding sites in six communities was one of the main risk factors for *S. haematobium* infection in residents [46].

The presence of *B. globosus* in our study areas was associated with certain behavioural activities observed at the HWCSs. Bathing and swimming/ playing significantly reduced the odds of finding *B. globosus* at HWCSs. This observation might be explained by the use of soap and the turbulent nature of these activities creating an environment less favourable for *B. globosus* [34]. In contrast, washing dishes and fishing significantly increased the odds of finding *B. globosus* at HWCS, perhaps indicating a nutrient rich environment caused by food remnants washed from dishes and indicated by the presence of fish, respectively. Interestingly, intermediate host snails infected with *S. haematobium* were significantly less likely to be present at HWCSs where water was collected for drinking and cooking. People not urinating and contaminating the water source that is used for potable water collection might be an explanation for this observation. Infected snails were also less present at HWCSs used for washing clothes. Soap might have an adverse effect on cercariae, as suggested elsewhere [17, 47].

In line with the baseline snail survey conducted at the onset of the SCORE operational research trial [14], but in contrast to studies previously conducted in Unguja [32, 35], water characteristics were not linked to the presence/ absence of *B. globosus* in the present study. Other, not presently measured factors and environmental dynamics, such as the permanence of the water body itself or flooding events that re-seed areas, might better predict the occurrence of intermediate host snails and merit future investigation.

We found that on average there were less taps in persistent hot-spot shehias than in low-prevalence shehias (*n* = 19 *vs n* = 30). In addition, the taps located in persistent hot-spot shehias had significantly lower odds of providing a constantly available water supply. While wells constituted a frequent and relatively constant water source particularly in the persistent hot-spot shehias of our study, they are reasonably cumbersome to use for children, who might use nearby freshwater bodies as alternatives for bathing or washing. The lack of reliable taps from which water collection is easy might contribute to people using potentially infectious freshwater bodies as a simple alternative for conducting domestic chores [48]. Improving and increasing the access to safe water and additional water, sanitation and hygiene (WASH) measures should be part of a sustainable schistosomiasis elimination strategy in Zanzibar and elsewhere [49–53].

Interestingly, in the “hottest” persistent hot-spot shehia, Koani, the distance from the school to the nearest HWCS (152 m) and HWCS containing *B. globosus* (218 m) was much shorter than the distance to the nearest SWS (376 m), although it was the shehia with the highest number of reliably working SWSs. Moreover, the highest number of *B. globosus* (*n* = 503) was collected in Koani.

Our small study design clearly limits the ability to attribute a meaning to unexpected findings. However, already the inclusion of only a small number of shehias has provided evidence that characteristics such as a larger number of HWCSs containing intermediate host snails and *B. globosus* infected with *S. haematobium*, a shorter distance from the primary school to the nearest HWCS and the lack of easy to use and reliably functioning SWSs play an important role in defining persistent hot-spot areas. This information can help to define, tailor and target future multidisciplinary interventions that will effectively reduce urogenital schistosomiasis transmission hot-spots in Zanzibar.

Recent reviews and analyses of the existing literature have outlined snail control as the most effective way of reducing schistosomiasis prevalence in endemic areas [54, 55]. Indeed, to sustainably curtail schistosomiasis transmission in Zanzibar, large-scale mollusciciding in hot-spot areas and focussed to HWCSs will be essential. With regard to the movement of potentially infected individuals between the shehias and the potential for re-contamination of treated freshwater bodies, it will be important to identify all HWCSs and the intermediate host snail abundance in areas of high transmission and to rigorously treat HWCSs regularly when intermediate host snails are present. The molluscicide niclosamide is the only commercially available and approved chemical for control of freshwater snails. However, while it is effective, it does have an impact on other aquatic organisms such as fish and amphibia and should be used carefully.

In addition to PC and area-wide snail control, the reduction of human-water contact by improving access to easy to use and reliably working SWSs and the minimisation of water contamination by changing human behaviour in hot-spot areas will be crucial for reaching elimination of transmission. Partnering with organisations and ministries that have the infrastructure and expertise to support and enhance WASH and educational measures can strengthen future interventions for elimination of urogenital schistosomiasis transmission in Zanzibar and elsewhere.

## Conclusion

The investigated persistent hot-spots in Zanzibar were characterized by a larger number of human-water contact sites containing intermediate host snails and *B. globosus* infected with *S. haematobium*, a shorter distance from the primary school to the nearest human-water contact site and the lack of easy to use and reliably functioning safe water sources. Rigorous focal mollusciciding or alternative snail control measures at all human-water contact sites near schools and villages, increasing access to safe water and sanitation, and enhanced behaviour change and health communication measures are needed to reduce prevalences in persistent hot-spot areas in order to reach elimination of schistosomiasis transmission.

## Additional file

Additional file 1: Results of univariable and multivariable analyses. (XLS 49 kb)

## Abbreviations

CDD: Community drug distributor
CI: Confidence interval
CWT: Community-wide treatment
HWCS: Human-water contact site
OR: Odds ratio
PC: Preventative chemotherapy
SCAN: Schistosomiasis Collection at the Natural History Museum
SCORE: Schistosomiasis Consortium for Operational Research and Evaluation
SWS: Safe water source
WASH: Water, sanitation and hygiene
WHO: World Health Organization
ZEST: Zanzibar Elimination of Schistosomiasis Transmission
